# From heart failure and kidney dysfunction to cardiorenal syndrome: TMAO may be a bridge

**DOI:** 10.3389/fphar.2023.1291922

**Published:** 2023-11-21

**Authors:** Jialun Zhang, Peining Zhu, Siyu Li, Yufei Gao, Yue Xing

**Affiliations:** ^1^ Department of Cardiology, The Second Hospital of Jilin University, Changchun, Jilin, China; ^2^ China-Japan Union Hospital of Jilin University, Changchun, Jilin, China

**Keywords:** trimethylamine oxide, heart failure, chronic kidney disease, cardiorenal syndrome, trimethylamine

## Abstract

The study of trimethylamine oxide (TMAO), a metabolite of gut microbiota, and heart failure and chronic kidney disease has made preliminary achievements and been summarized by many researchers, but its research in the field of cardiorenal syndrome is just beginning. TMAO is derived from the trimethylamine (TMA) that is produced by the gut microbiota after consumption of carnitine and choline and is then transformed by flavin-containing monooxygenase (FMO) in the liver. Numerous research results have shown that TMAO not only participates in the pathophysiological progression of heart and renal diseases but also significantly affects outcomes in chronic heart failure (CHF) and chronic kidney disease (CKD), besides influencing the general health of populations. Elevated circulating TMAO levels are associated with adverse cardiovascular events such as HF, myocardial infarction, and stroke, patients with CKD have a poor prognosis as well. However, no study has confirmed an association between TMAO and cardiorenal syndrome (CRS). As a syndrome in which heart and kidney diseases intersect, CRS is often overlooked by clinicians. Here, we summarize the research on TMAO in HF and kidney disease and review the existing biomarkers of CRS. At the same time, we introduced the relationship between exercise and gut microbiota, and appropriately explored the possible mechanisms by which exercise affects gut microbiota. Finally, we discuss whether TMAO can serve as a biomarker of CRS, with the aim of providing new strategies for the detection, prognostic, and treatment evaluation of CRS.

## 1 Introduction

Cardiorenal syndrome (CRS) is commonly encountered in clinical practice and includes a range of diseases involving the heart and kidneys. This condition occurs when one organ is acutely or chronically dysfunctional, often resulting in other organ dysfunction as well. This syndrome was first described in 1836 by Robert Bright who observed that patients with advanced kidney disease often experience significant changes in the structure of the heart (Bright, 1836). Many studies on heart and kidney diseases have brought the association between the two organs even closer, particularly in terms of hemodynamics, pathophysiology, and treatment. Heart failure (HF) and renal disease are sometimes mutually causative. Both HF and CKD involve the activation of the neuroendocrine system and interference from inflammatory cytokines, ultimately leading to structural impairing in the heart or kidneys. HF and CKD are the keys to unlocking the study of CRS. Researchers can gain a deeper understanding of CRS by thoroughly understanding the development of HF and CKD. Recently, numerous scholars have discovered the relationship between the TMAO and a poor prognosis in HF and CKD (Tang et al., 2015a; Kuehn, 2019). Thus, TMAO is a potential biomarker for evaluating the prognosis of HF and CKD (Chioncel and Ambrosy, 2019; Zixin et al., 2022). In this article reviews the progress in research on the involvement of TMAO in HF, CKD, and CRS, and review the possible existing biomarkers of CRS. Finally, we discuss the potential role of TMAO in CRS and whether TMAO can serve as a biomarker of CRS.

## 2 Production and metabolism of TMAO

**FIGURE 1:**
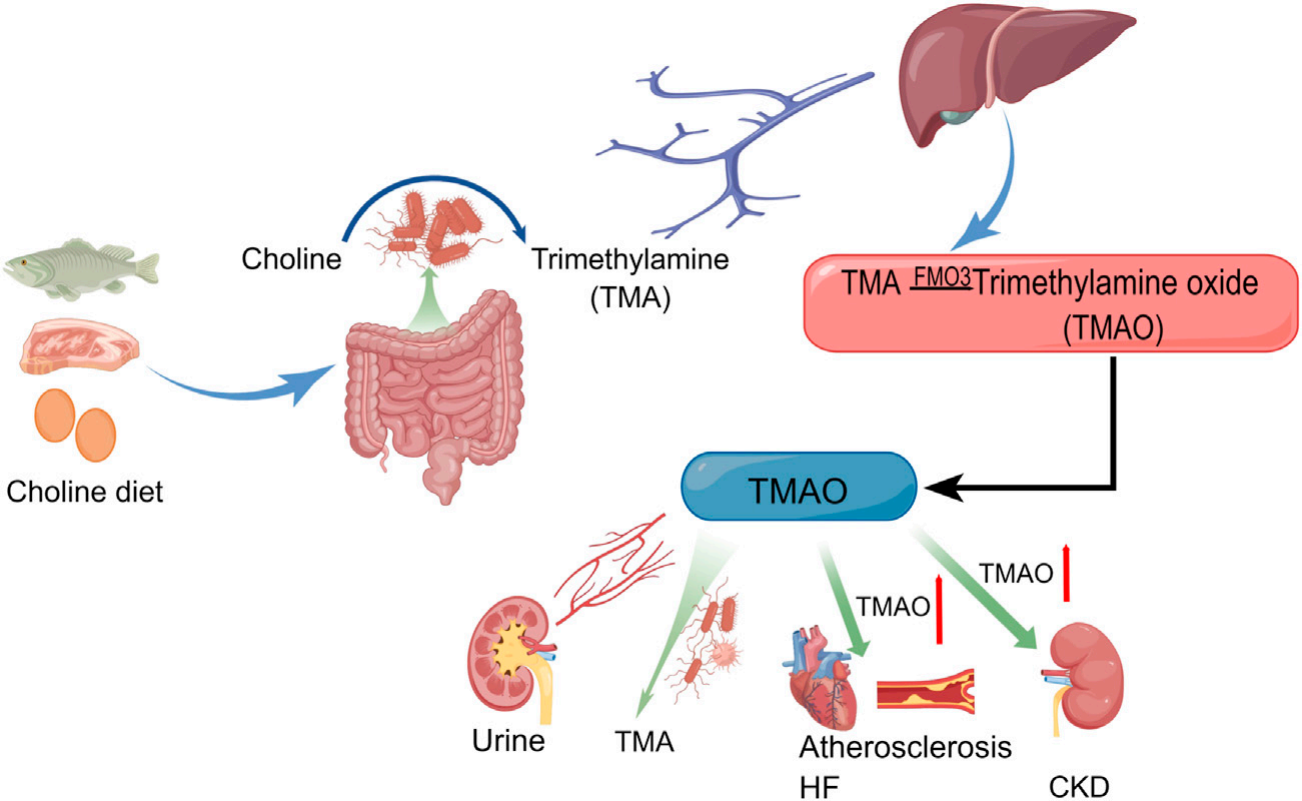
Production and metabolism of TMAO (By Figdraw).

TMAO is formed by the transformation of TMA in the liver, and this process can not be separated from the flavin containing monooxygenase 3 (FMO3) (Lang et al., 1998; Wang et al., 2011; Ufnal et al., 2015). TMA mainly comes from dietary intake of meat, egg yolks and so on that are metabolized by gut bacteria to produce TMA (Subramaniam and Fletcher, 2018; Janeiro et al., 2018). In adults, the gut microbiota are primarily composed of the phyla Bacteroidetes and Firmicutes (Cani, 2018). The bacteria that produce TMA include *Clostridium*, *Proteus*, *Shigella*, and *Aeromonas* (Barrett and Kwan, 1985; Salazar et al., 2020; Li J. et al., 2022). Although TMA is a natural component of human blood, urine and cerebrospinal fluid, the content of TMA is extremely low (Dorée and Golla, 1911). It should be noted that TMAO generated from the conversion of TMA is a uremic toxin closely associated with the pathophysiology of a variety of heart and kidney diseases (Xiong et al., 2023). TMAO is mostly excreted in the urine (Al-Waiz et al., 1987; Smith et al., 1994; Lang et al., 1998; Tomlinson and Wheeler, 2017), and a small amount spontaneously degrades back to TMA at a slow rate (Jia et al., 2020). TMAO levels in plasma cannot be separated from diet, and plasma TMAO levels can be lowered within 4 weeks of stopping red meat consumption (Wang et al., 2019). The production, metabolism, and pathogenic pathways of TMAO are shown in Figure 1.

## 3 TMAO and HF

Gut microbiota and its metabolites are involved in the pathogenesis of HF (Kuehn, 2019). The intestinal leakage hypothesis suggests that the reduction of cardiac output and the increase of systemic congestion will cause intestinal mucosal ischemia and edema, which will further lead to the increase of intestinal permeability to proinflammatory mediators (Zhang et al., 2015; Anderson et al., 2022). The disturbance of intestinal secretion and metabolism is mainly manifested in the imbalance of intestinal flora and the changes of metabolic derivatives TMA and TMAO. There is a significant difference in the distribution of the gut microbiota metabolite TMAO in patients with HF, which may indicate a poor long-term prognosis for HF (Chioncel and Ambrosy, 2019). In 2014, Tang et al. (2014) measured plasma TMAO concentrations in 720 patients with CHF. They found that, compared to HF patients with lower plasma TMAO levels, HF patients with higher plasma TMAO levels had a significantly increased risk of long-term mortality. Tang et al. (2015b) included 112 patients with HF and measured their plasma TMAO levels using mass spectrometry. The increase of plasma TMAO concentration was positively correlated with left ventricular diastolic dysfunction. Trøseid et al. (2015) conducted a study that included 155 patients with CHF as the experimental group, 100 stable coronary artery disease (CAD) patients without HF as the experimental group, 33 randomly selected normal individuals as reference. They measured the plasma TMAO levels of all study subjects and found that an increase in TMAO concentration was significantly associated with an increased risk of cardiovascular events, especially in patients with HF. The increase in plasma TMAO concentration in patients with acute HF indicates a poor 1-year prognosis. After excluding the interference of renal factors, TMAO may become an independent indicator for predicting death/recurrent HF (Suzuki et al., 2016). Organ et al. (2016) used a murine model and provided the experimental group of mice with a choline diet or a diet containing TMAO. Compared to the control group, the severity of HF in the experimental group of mice was significantly increased. In 2018, Hayashi et al. (2018) checked the plasma TMAO concentrations in 22 patients with acute HF. The plasma TMAO titer in patients with acute HF was elevated compared to that of the control group participants. In the same year, Cui et al. (2018) found that microbial genes related to TMAO production were significantly upregulated in patients with CHF. Huc et al. (2018) established a mouse model and provided the experimental group of animals with TMAO-containing water; the control group was given water without TMAO. They measured the plasma TMAO concentration in the experiment and control mice and found that a mildly elevated in plasma TMAO levels ameliorated diastolic dysfunction and cardiac fibrosis in the rats of the experimental group. This result showed that elevated plasma TMAO concentration was negatively correlated with increased risk of cardiovascular rehospitalization or death, a finding that may contradict existing findings. Suzuki et al. (2019) measured plasma TMAO concentrations in patients included in the BIOlogy Study to TAilored Treatment in Chronic HF (BIOSTAT-CHF), a systems biology study of individualized treatment for CHF, and confirmed that high concentration of TMAO in plasma was closely related to adverse events (Karlin et al., 2019). Another mouse model study (Li et al., 2019) demonstrated that TMAO induces cardiac fibrosis through the Smad3 signaling pathway. The findings of this study suggest that interfering with the production of TMAO may be a target for preventing and treating cardiac hypertrophy. In 2020, Guo et al. (2020) found that plasma TMAO levels are closely related to HF with preserved ejection fraction (HFpEF) and renal function. Thus, TMAO may become a new diagnostic biomarker for heart failure and renal impairment. In the same year, Li et al. (2020) searched for and analyzed published studies in electronic databases (PubMed and EMBASE) on the relationship between plasma TMAO concentration and all-cause mortality in adult patients with HF. They found that renal dysfunction might be one of the factors associated with high plasma TMAO concentration and poor prognosis in patients with HF. In 2021, Dong et al. (2021) recruited 61 patients with HFpEF patients as controls and found that plasma TMAO levels were highly correlated with the risk of HFpEF. Kinugasa et al. (2021) found that high levels of TMAO in plasma during hospitalization in patients with HFpEF were positively correlated with the risk of subsequent cardiovascular events after discharge. Emoto et al. (2021) performed a genomic analysis of the intestinal bacteria and found that plasma TMAO concentration in HF patients were related to the abundance of carnitine oxygenases A/B (cntA/B). A study involving 806 patients with acute HF found that the plasma TMAO concentration in patients with acute HF was adverse outcomes (Israr et al., 2021). Interestingly, Yuzefpolskaya et al. (2021) found that TMAO levels remained elevated in the long term treatment of patients with HF who underwent left ventricular assist device (LVAD) implantation or heart transplantation. Researchers believe that TMAO levels are not related to inflammation, endotoxemia, oxidative stress, or gut dysbiosis (Yuzefpolskaya et al., 2021). In 2022, Li N. et al. (2022) found that plasma TMAO levels were significantly elevated in patients with acute myocardial infarction (AMI) who subsequently developed HF, and that elevated plasma TMAO levels were independently keeping in touch with poor prognosis. A study on the correlation between brown fat metabolism disorders and HF found that TMAO can aggravate HF by inhibiting the activity of mitochondrial complex IV in myocardial tissue and reducing ATP and phosphocreatine (Yoshida et al., 2022). Recently, a study (Wei et al., 2022) found that circulating TMAO levels were influenced by the SNP rs2266782 in the FMO3 gene, and elevated levels of plasma TMAO may indicate an increased risk of various deaths. This increased risk of cardiovascular death caused by elevated plasma TMAO levels was independent of other potential confounding factors (Wei et al., 2022; Qiu et al., 2022). In conclusion, the relationship between TMAO and an adverse prognosis in HF is not yet clear, and different results have been recorded in published clinical studies. A recent study (Li X. et al., 2022) was shown that patients with high plasma concentration of TMAO had an increased risk of mace and all-cause mortality compared with patients with low plasma concentration of TMAO. In recently, research on the relationship between HF and TMAO has entered a critical stage, and most evidence suggests the relationship between elevated circulating TMAO levels and adverse events in HF. At present, the causality and specific mechanism of increased circulating TMAO concentration leading to increased cardiovascular events are attracting researchers’ interest, including racial differences, dietary differences, changes in gut microbiota, and drugs. Reducing the intake of foods rich in carnitine and choline seems to be highly consistent with the low-salt, low-fat diet advocated for cardiovascular disease. However, diet does not seem to be as important in patients with decompensated HF, especially in cases of gastrointestinal congestion. The relationships between TMAO and human studies or animal HF models are presented in Table 1.

**TABLE 1:** TMAO and human or animal HF models.

Models	Diseases	TMAO	MACEs	All-cause mortality	Other outcomes	References
Human	CHF	Higher plasma TMAO levels	-	Increase	-	Suzuki et al. (2016)
	CHF	Higher plasma TMAO levels	-	Increase	-	Organ et al. (2016)
	CHF	Higher plasma TMAO levels	-	Increase	-	Hayashi et al. (2018)
	AHF	Higher plasma TMAO levels	-	Increase	-	Cui et al. (2018)
	CHF	Lower TMAO levels	-	-	With favorable outcome regardless of treatment	Guo et al. (2020)
	HFpEF	Higher plasma TMAO levels	-	-	Increased HFpEF risk	Israr et al. (2021)
	AHF	Higher plasma TMAO levels	Increase	Increase	-	Yuzefpolskaya et al. (2021)
	AHF	Higher plasma TMAO levels	Increase	Increase	-	Yoshida et al. (2022)
	AHF	Higher plasma TMAO levels	Increase	-	-	Qiu et al. (2022)
	CHF	Higher plasma TMAO levels	Increase	Increase	-	Bain et al. (2006)
Mouse	HF	Diet containing TMAO	-	-	Heart failure severity	Huc et al. (2018)
	HF	Low-dose TMAO	-	-	Reduces diastolic dysfunction and heart fibrosis	Li et al. (2019)
	HF	Higher plasma TMAO levels	-	-	Induces cardiac hypertrophy and fibrosis	Dong et al. (2021)

HF, heart failure; AHF, acute heart failure; CHF, chronic heart failure; MACEs, major adverse cardiovascular events; HFpEF, heart failure with preserved ejection fraction.

## 4 TMAO and CKD

Bain et al. (2006) measured the concentrations of TMA and TMAO in 20 plasma samples using gas chromatography-mass spectrometry, including 10 healthy controls and 10 subjects with CKD. They found that the TMA and TMAO levels in patients with CKD increased during hemodialysis. In 2015, Tang et al. (2015a) followed up with 521 stable CKD patients and measured TMAO and found that elevated plasma TMAO was positively correlated with long-term mortality risk and could promote disease progression in CKD patients. Hai et al. (2015)’s study showed that ESRD patients receiving chronic hemodialysis before dialysis had significantly higher TMAO concentrations than the control group, and there was no difference in TMAO and creatinine clearance rates between these groups during dialysis. Fogelman (2015) pointed out that TMAO has a dual identity, on the one hand, it can be used as a biomarker, on the other hand, it can exist as a toxin. In 2016, a study found for the first time a correlation between the FMO3 SNP genotype at amino acid position 158 and both plasma TMAO concentration and all-cause mortality (Robinson-Cohen et al., 2016). However, after adjusting for potential confounding factors, no significant correlation was found between the TMAO levels and clinical outcomes in patients with CKD. Sick patients with CKD showing a worsening renal function have higher plasma TMAO levels; however, after kidney transplantation, plasma TMAO levels return to normal (Missailidis et al., 2016). Kidney transplantation can reduce plasma or serum TMAO levels, but to a lesser extent than other renal indices (Poesen et al., 2016; Stubbs et al., 2016). It is not clear whether reducing TMAO levels can dilute the risk of cardiovascular events in CKD patients (Kim et al., 2016). In experimental mice fed a high fat diet (HFD), circulating TMAO levels were significantly elevated, which promoted oxidative stress and inflammation in the kidneys, leading to kidney damage (Sun et al., 2017). In 2016, Al-Obaide et al. (2017) found that TMA were more prevalent in patients with type 2 diabetes mellitus (T2DM) and late-stage CKD than that in healthy adults and that the concentration of TMAO in the serum of T2DM-CKD patients was significantly higher than that of the healthy control group (Al-Obaide et al., 2017). Xu et al. (2017) transferred fecal samples from CKD patients and healthy controls to antibiotic treated c57bl/6 mice. Compared with the control group, the plasma TMAO level of mice transplanted with intestinal microbiota of CKD patients was significantly increased. There is a complex intimate relationship between elevated plasma TMAO concentration and renal dysfunction and intestinal microbiota Dysbiosis in CKD patients. At the same time, many researchers have shown that TMAO has the potential as a biomarker for CKD and a target for diagnosis and treatment (Tomlinson and Wheeler, 2017). In 2018, Li et al. (2018) found that the concentration level of TMAO in the circulation of CKD rats was higher than that of control rats, and this may promote CKD-related cardiovascular diseases through vascular oxidative stress and inflammation. Johnson et al. (2018) used a murine model and found that increased hepatic monooxygenase activity was associated with elevated circulating TMAO levels. Circulating TMAO levels derived from dietary sources are positively correlated with CKD progression, while increased gut microbiota and TMAO derivatives are risk factors for cardiovascular events in patients with CKD (Castillo-Rodriguez et al., 2018; Lau et al., 2018; Li and Tang, 2018). In 2019, Pignanelli et al. (2019) found that patients with reduced glomerular filtration rate (EGFR) had significantly higher plasma TMAO levels. An *in vitro* FMO enzyme activity assay in mice suggested that non-dietary activation of FMO-mediated TMAO production may be a new biochemical mechanism (Prokopienko et al., 2019). Patients with decreased renal function, is a high degree of correlation between plasma or serum TMAO levels and eGFR values (Pelletier et al., 2019; Jia et al., 2019). A study by Stubbs et al. (2019) involving 1,243 people found that baseline serum TMAO levels were not significantly associated with MACEs or stroke. However, among patients undergoing routine hemodialysis, high TMAO levels are significantly associated with higher hospitalization rates, and patients with high plasma TMAO levels have higher hospitalization rates than those with low plasma TMAO levels (Zheng et al., 2020). However, the accuracy of measuring TMAO levels in circulation through enzyme-linked immunosorbent assay is difficult to estimate. Recent studies have found that circulating TMAO can promote vascular calcification in rats with CKD and activates the NLRP3 inflammasome and NF-κB signaling pathways (Zhang et al., 2020). In CKD mouse models, the inhibition of TMAO production can decrease tubulointerstitial fibrosis and functional damage (Gupta et al., 2020). In patients undergoing cardiovascular surgery, high levels of serum TMAO are associated with the progression of CKD and the number of coronary artery infarction (Mafune et al., 2016). Given the close relationship between TMAO and CKD, the rapid detection of gut microbiota metabolites, such as TMAO, can better prevent cardiovascular CKD and its associated events (Chang et al., 2021). Zhang W. et al. (2021) established a mouse model and observed that the inhibition of TMAO production alleviated CKD in mice. Kapetanaki et al. (2021) revealed that TMAO promotes human renal fibroblast fibrosis through the PERK/Akt/mTOR, NLRP3, and caspase-1 signaling pathways, laying the foundation for further elucidation of the molecular mechanism of TMAO production. In cell line models of CKD, TMAO-mediated nuclear translocation of Y-box binding protein-1 directly downregulates Gadd45a expression to promote cell cycle progression (Wang L. et al., 2021). A significant increase in circulating TMAO concentration is associated with the risk of death in patients with CKD; However, this relationship may depend on TMAO dose (Zeng et al., 2021). Human intervention in TMAO can reduce the risk of cardiovascular disease in CKD patients (Ravid et al., 2021). In renal excision-induced CKD rat studies, TMAO was observed to activate p38 phosphorylation and the expression of human antigen R, thereby elevating the inflammatory factors MCP-1, TNF-α, IL-6, IL-1β, and IL-18. At the same time, the researchers also confirmed that TMAO can accelerate the formation process of NLRP3 inflammasome in kidney tissue and the activation of lytic caspase-1 and IL-1β, once again proving that the results of Kapetanaki et al. are reliable (Lai et al., 2022). Serum TMAO levels and the ability of protein consumption (protein energy wasting, PEW) was significantly associated with the prevalence of protein energy wasting (PEW) in maintenance hemodialysis patients (MHD) (Hu C. et al., 2022). There is a positive correlation between serum TMAO levels and brachial ankle pulse wave velocity (baPWV) in CKD patients (Hsu et al., 2022). One study (Zhou et al., 2022) showed that circulating high levels of TMAO were associated with an increased risk of long-term mortality in patients with CKD; However, this relationship may depend on the dose of TMAO and not on renal function. The results of this research differed from those of several other studies. TMAO produced by intake of diet rich in choline and carnitine can reduce kidney mass and increase α Expression of smooth muscle actin. In addition, disrupting the gut microbiota while inhibiting the TMAO biosynthetic pathway mitigates kidney damage in mouse models of CKD. TMAO not only promoted the increased risk of death in CKD patients, but also increased the incidence of peritonitis in peritoneal dialysis patients (Zhang et al., 2022). The elucidation of mechanisms by which TMAO aggravates kidney disease injury seems to have made preliminary progress, laying the foundation for a more comprehensive description of the molecular mechanisms by which TMAO causes kidney disease. Whether TMAO can be used as a new target to improve the prognosis of CKD requires more studies (Meijers et al., 2019). Research related to TMAO and human or animal CKD is summarized in Table 2.

**TABLE 2:** Research related to TMAO and human or animal chronic kidney disease models.

Models	Diseases	TMAO	MACEs	All-cause mortality	Other outcomes	References
Human	CKD	Higher plasma TMAO levels	-	Increase	-	Tang et al. (2015a)
	CKD	Higher plasma TMAO levels	-	Increase	-	Robinson-Cohen et al. (2016)
	CKD	Higher plasma TMAO levels	-	Increase	-	Missailidis et al. (2016)
	CKD	Higher Serum TMAO levels	-	-	Strong inverse association with eGFR	Stubbs et al. (2016)
	CKD	Higher plasma TMAO levels	-	Increase	-	Kim et al. (2016)
	ESKD	Higher Serum TMAO levels	-	-	Serum TMAO was not associated with cardiovascular outcomes	Stubbs et al. (2019)
	CKD	Higher plasma TMAO levels	-	-	Increased incidence of inpatient events	Zheng et al. (2020)
	CKD	Higher Serum TMAO levels	-	-	Body mass index (BMI) and dietary protein intake decreased	Hu et al. (2022a)
	CKD	Higher Serum TMAO levels	-	-	Serum TMAO was positively correlated with C-reactive protein level and either left or right baPWV	Hsu et al. (2022)
	CKD	Higher Serum TMAO levels	-	-	Exacerbate peritoneal inflammation	Zhang et al. (2022)
Mouse	CKD	Higher plasma TMAO levels	-	-	Promote renal oxidative stress and inflammation	Sun et al. (2017)
	CKD	Higher plasma TMAO levels	-	-	Contributes to endothelial dysfunction	Li et al. (2018)
	CKD	Inhibition of TMAO production	-	-	Reduces renal tubulointerstitial fibrosis and functional impairment	Gupta et al. (2020)
	CKD	Inhibition of TMAO production	-	-	Attenuate CKD development	Zhang et al. (2021a)

ESKD, end-stage kidney disease; CKD, chronic kidney disease; baPWV, brachial-ankle pulse wave velocity.

## 5 CRS and biomarkers

### 5.1 Existing biomarkers in CRS

The initial definition of CRS was that it is the outcome of interactions between the renal and other circulatory compartments that increased circulating volume, exacerbated symptoms, and disease progression of patients with HF (Rangaswami et al., 2019). In 2008, Ronco et al. (2008) classified the CRS phenotype into five subtypes according to the severity of the disease and the involvement of secondary organs. The establishment of the definition and classification of CRS has greatly promoted the early identification of patients with CRS, helping clinicians better diagnose and manage CRS (House et al., 2010). In 2021, Zhang Y et al. (2021) proposed a sixth CRS subtype. They further divided traditional secondary CRS into acute and chronic secondary CRS, naming the former type 5 CRS and the latter type 6 CRS.

In order to explore whether TMAO can serve as a biomarker for CRS, we cannot ignore the existing biomarkers for CRS. The biomarkers related to the diagnosis and prognosis of CRS include both cardiac and renal markers. Here, we described the different biomarkers in detail of the heart and kidneys in CRS. BNP and NT-proBNP are widely used in the diagnosis and exclusion of HF in clinical practice (Castiglione et al., 2022; Heidenreich et al., 2022). Compared with AHF patients without renal impairment, the baseline BNP level of CKD patients with HF was significantly higher than that of CKD patients alone; BNP levels were significantly higher in patients with evidence of CRS (Ronco et al., 2008; House et al., 2010; Palazzuoli et al., 2014; Rangaswami et al., 2019; Zhang Y. et al., 2021; Castiglione et al., 2022; Heidenreich et al., 2022). Studies have found that sST2 in combination with BNP/NT-proBNP can better predict the occurrence of HF adverse events, and sst2 is not affected by renal function, so it has potential value for the prognosis of CRS (Yancy et al., 2017; Chirinos et al., 2020). Galectin-3 has been shown to be involved in pathological cardiac fibrosis and remodeling (Suthahar et al., 2018). Elevated plasma galectin-3 levels were associated with renal dysfunction and were an independent predictor of all-cause mortality in HF (Lok et al., 2010; Tang et al., 2011; Chen et al., 2020). Elevated troponin levels are positively correlated with the risk of cardiovascular death (Meijers et al., 2021; Chapman et al., 2020). Whether myocardial injury is due to cardiac dysfunction or renal insufficiency, cardiac troponin has reference value for the prognosis assessment of CRS (Gunsolus et al., 2018; Yan et al., 2020; Berg et al., 2022).

Serum creatinine is a common indicator used to evaluate kidney function; however, it has limited value in assessing acute renal dysfunction and uremia (Levey et al., 1988; Kashani et al., 2020). Despite the limitations of serum creatinine in evaluating kidney function, it can still be used for the diagnosis and prognostic assessment of CRS in patients with renal dysfunction and heart injury (Rangaswami et al., 2019; Han et al., 2020). CysC level and proteinuria are biomarkers of glomerular filtration and integrity in CRS (Zhang et al., 2011; Rangaswami et al., 2019; Christiadi et al., 2022; Huang et al., 2022). CysC has a good predictive value for renal dysfunction in patients with acute HF CRS1 patients (Lassus et al., 2007). CysC combined with NT-proBNP levels can better predict the risk of HF in CKD patients (Wang S. et al., 2021). Reducing of urinary albumin is a biomarker of early improvement in T2-CRS after CRT (Gala-Błądzińska et al., 2020). Funahashi et al. (2022) suggested that improving CRS-1 outcomes might be possible through albumin resuscitation. MI-induced CRS in rats accelerates glomerular remodeling and the production of trace albuminuria (Dong et al., 2015). The predictive model combining plasma NGAL and creatinine D1 on the first day of admission had high accuracy in predicting CRS1 (Phan Thai et al., 2020). The combination of NGAL and NT proBNP in serum has early diagnostic value for type 1 CRS. Researcher showed that serum NGAL and NT-proBNP independently had a high predictive value for CRS (Song et al., 2021). The research data showed that elevated urine NGAL levels may serve as an early detection marker for left HF-related kidney injury (Entin-Meer et al., 2012). Alvelos et al. (2011) found that the critical value of NGAL was 170 ng/L and the sensitivity of NGAL in diagnosing type 1 CRS was 100%. These findings suggest that KIM-1 is a potential biomarker of CRS with an additional prognostic value (Jungbauer et al., 2011). A report has identified the progressive changes in renal KIM-1 expression in a rat model of myocardial infarction over time (Lekawanvijit et al., 2012). IL-18 levels in the serum of patients with CRS type 1 were significantly higher than those in patients with AHF, The CRS type 1 group had significantly higher levels of IL-18, IL-1, and MPO than did the AHF group. Patients with CRS type 1 present with elevated levels of pro-inflammatory cytokines and oxidative stress markers, elevated levels of tissue injury markers, and decreased levels of hemoglobin, all of which may be related to the pathophysiology of CRS type 1 (Virzì et al., 2018). In a prospective multi-center study, researchers measuring uAGT at the time of AKI diagnosis in acute HF patients with AHF helps identify patients with CRS with the highest risk of adverse outcomes (Chen et al., 2016). The existing biomarkers of CRS have been summarized in Table 3.

**TABLE 3:** Biomarkers of the heart and kidney in CRS.

Biomarkers	Function of biomarker	Diagnostic value	Prognostic value	References
BNP	Marker of HF	-	CRS	Palazzuoli et al. (2014); Rangaswami et al. (2019)
sST2	Marker of cardiac fibrosis	-	CRS	Yancy et al. (2017); Chirinos et al. (2020)
Galectin-3	Marker of cardiac fibrosis	-	CRS	Lok et al. (2010); Tang et al. (2011); Rangaswami et al. (2019); Chen et al. (2020)
hs-cTnI	Marker of cardiac myocyte injury	-	CRS	Gunsolus et al. (2018); Rangaswami et al. (2019); Chapman et al. (2020); Yan et al. (2020); Berg et al. (2022)
Serum creatinine	Marker of kidney injury	CRS	CRS	Rangaswami et al. (2019); Han et al. (2020)
CysC	Marker of GFR	CRS	CRS	Lassus et al. (2007); Wang et al. (2021b); Christiadi et al. (2022)
Albuminuria	Marker of glomerular injury	CRS	CRS	Dong et al. (2015); Funahashi et al. (2022)
NGAL	Marker of CRS 1	-	CRS	Alvelos et al. (2011); Entin-Meer et al. (2012); Song et al. (2021)
KIM-1	Marker of early kidney injury	-	CRS	Jungbauer et al. (2011); Lekawanvijit et al. (2012)
IL-18	Marker of acute kidney injury	-	CRS	Virzì et al. (2018)
UAGT	Marker of acute kidney injury	-	CRS	Chen et al. (2016)

BNP, brain natriuretic peptide; sST2, soluble suppressor of tumorigenicity; hs-cTnI, high-sensitivity cardiac troponin I; CRS, CRS; CysC, cystatin C; NGAL, neutrophil gelatinase-associated lipocalin; KIM-1, kidney injury molecule-1; UAGT, urine angiotensinogen.

### 5.2 Research on TMAO as a biomarker for CRS

TMAO has made some progress in the study of heart failure and renal dysfunction. The research on CRS and TMAO has just begun. Hu DY. et al. (2022) used liquid chromatography-tandem mass spectrometry (LC-MS/ms) to analyze plasma samples from patients with uremia and healthy controls and found that TMAO is a promising uremia biomarker. On one hand, TMAO can promote renal fibrosis; on the other hand, its titers increase with the worsening of CKD, leading to a doubling of heart and kidney damage (Taguchi et al., 2019). Zou et al. (2021) revealed the relationship between circulating TMAO and CRS2 in rats. Compared to controls, the experimental rats received the TMAO inhibitor treatment and did not show aggravated heart or kidney dysfunction. The heart and kidney indicators of the experimental rats that received the TMAO inhibitor treatment did not deteriorate significantly. These findings suggest that the attenuation of circulating TMAO levels can improve heart and kidney damage and prevent the progression of CRS2. Another study showed that TMAO is a newly identified uremic toxin that can reduce the risk of cardiovascular disease in CKD patients (Ravid et al., 2021; Xiong et al., 2023). The ratio of higher plasma and lower urinary solute acid concentrations to plasma concentrations was independently associated with cardiovascular and all-cause mortalities in patients with diabetic kidney disease. The association between the ratio of urinary to plasma concentrations and mortality suggests a relationship between the clearance rate of uremic solutes and the pathogenesis of cardiovascular disease (Sapa et al., 2022). In addition, giving CKD mice a choline diet can inhibit the protein levels of Hif-1α in the hearts of CKD mice, and the Hif-1α stabilizer FG-4592 can improve heart vascular development and functional impairment in CKD mice fed a high-choline diet. These data suggest that TMAO inhibits cardiac vascular development by reducing Hif-1α protein levels, ultimately exacerbating heart dysfunction in CKD mice (Xie et al., 2022). TMAO has established connections with both heart and kidney failure through both known and unknown pathways. However, research on biomarkers that directly link TMAO with CRS is rare. Therefore, we have to pay attention to the research progress of TMAO in heart failure and CKD, as well as the research status of other biomarkers of CRS.

## 6 Summary

CRS is a clinical syndrome involving complex pathophysiological pathways and dysfunction of both the heart and kidneys (McCallum and Sarnak, 2023). Recent studies have shown that CRS is associated with high morbidity and mortality in patients with acute or chronic HF or CKD (Kim et al., 2023). However, many clinicians have limited knowledge of CRS and often overlook its diagnosis in clinical practice, resulting in a low diagnostic rate of CRS patients. TMAO is a small molecule that promotes the progression of atherosclerosis through various pathways and is associated with the disease status of HF and CKD. TMAO exacerbates adverse events in patients with HF and CKD and may play an important role in the long-term prognostic assessment of HF and CKD. Despite the establishment of the American Heart Association’s diagnostic and treatment guidelines for CRS in 2019, further efforts are needed to improve clinicians’ understanding of CRS.

At present, many issues remain unclear in the research on TMAO and CRS. First, the causal relationship between changes in TMAO concentration and heart and kidney failure and CRS is uncertain. This requires more researchers and institutions to carry out multi-center cooperation to establish an extensive and comprehensive database to monitor the continuous changes in TMAO levels in normal people with different dietary environments, different races, and people with heart and kidney failure. Only then can the causal relationship between TMAO and heart and kidney failure and cardiorenal syndrome be clarified. Secondly, the clinical diagnosis of CRS has not been taken seriously. This requires calling on clinicians to pay attention to the identification and diagnosis of CRS, pay attention to the role of intestinal flora metabolites in the progression of CRS, and encourage more clinicians to actively join TMAO Research related to CRS. At present, the accurate determination of TMAO relies on expensive LC-MS/ms, which limits the development of related research. The accuracy of existing TMAO detection kits has not been widely recognized. Whether the test performance of affordable TMAO detection kits is consistent with that of LC-MS/ms requires more research to confirm.

Paying attention to changes in the gut microbiota derivative TMAO, we have to pay attention to the gut microbiota. Gut microbiota are similar to endocrine organs, and gut microbiota can receive stimulation and send signals. Appropriate exercise has beneficial effects on the gut microbiota, which may involve complex stress responses and neuroenteroendocrine activation (Clark and Mach, 2016). Appropriate exercise can stimulate the proliferation of beneficial bacteria and change the diversity of gut microbiota. These beneficial bacteria can produce short-chain fatty acids and other substances to regulate mucosal immunity and improve barrier function, while improving the Bacteroidetes/*Bacillus* phylum ratio (Wastyk et al., 2021). At present, we see more of the effects of exercise on gut microbiota and metabolites, but the complex pathophysiological relationships involved are unclear. This includes that exercise may interfere with intestinal microorganisms and metabolites, and then inhibit the activation of inflammatory signaling pathways such as NLRP, thereby hindering the progression of related diseases (Quiroga et al., 2020). Secondly, exercise may affect the gut’s own interoceptive circuitry and subsequently affect brain neuronal activity. Exercise may deplete the corresponding metabolites of intestinal microorganisms. The signal is transmitted to special neurons in the brain through the brain-gut axis and stimulates the secretion of endogenous reward substances. Positive feedback promotes exercise ability. Finally, exercise may trigger a stress response that activates the sympathetic-adrenal medulla and hypothalamic-pituitary-adrenal (HPA) axes, resulting in the conduction of electrochemical signals and ultimately the release of cytokines and other small cells that facilitate signaling molecular. Appropriate exercise can change the abundance of some gut microbiota and the levels of derivatives such as TMAO through a series of complex neuroendocrine activation and signal transduction. This may be a new approach to intervene in the relationship between TMAO and heart and kidney failure, as well as the progression of cardiorenal syndrome. TMAO has great potential as a biomarker for evaluating the prognosis of CRS. Of course, more evidence is needed.

Many researchers view TMAO as a new marker for assessing the prognosis of patients with HF and CKD, and its potential role in CRS diagnosis and prognosis cannot be underestimated. As more scholars conduct in-depth research, TMAO has the potential to serve as a valuable biomarker for the diagnosis and long-term prognostic evaluation of CRS patients.

## 7 Clinical significance and prospects

The gut microbiota and metabolite TMAO serve as a bridge connecting heart, kidney failure, and CRS. At present, there are certain limitations in evaluating the diagnostic and prognostic value of CRS using diagnostic/prognostic markers for heart failure or chronic kidney disease. If TMAO can serve as a biomarker for CRS, it will greatly improve the diagnosis and prognosis evaluation of CRS by clinical doctors.

As a new uremic toxin and potential biomarker, TMAO is involved in changes in the heart-gut-kidney axis, including neuroendocrine activation, hemodynamic changes, alterations in gut microbiota, and intestinal congestion (Xiong et al., 2023). Many researchers view TMAO as a new biomarker for evaluating the prognosis of patients with heart failure and chronic kidney disease, and its potential role in the diagnosis and prognosis of CRS will also receive more attention. With the in-depth research of more and more scholars, TMAO has great potential as a valuable biomarker for the diagnosis and long-term prognosis evaluation of CRS patients.
